# Posterior Tibial Nerve Stimulation in Fecal Incontinence: A Systematic Review and Meta-Analysis

**DOI:** 10.32598/bcn.9.10.290

**Published:** 2019-09-01

**Authors:** Arash Sarveazad, Asrin Babahajian, Naser Amini, Jebreil Shamseddin, Mahmoud Yousefifard

**Affiliations:** 1. Colorectal Research Center, Iran University of Medical Sciences, Tehran, Iran.; 2. Liver and Digestive Research Center, Kurdistan University of Medical Sciences, Sanandaj, Iran.; 3. Cellular and Molecular Research Center, Iran University of Medical Sciences, Tehran, Iran.; 4. Molecular Medicine Research Center, Hormozgan Health Institute, Department of Parasitology, Faculty of Medicine, Hormozgan University of Medical Sciences, Bandar Abbas, Iran.; 5. Physiology Research Center, Faculty of Medicine, Iran University of Medical Sciences, Tehran, Iran.

**Keywords:** Fecal incontinence, Tibial nerve, Electrical nerve stimulation, Tibial neuromodulation

## Abstract

**Introduction:**

The present systematic review and meta-analysis aims to investigate the role of Posterior Tibial Nerve Stimulation (PTNS) in the control of Fecal Incontinence (FI).

**Methods:**

Two independent reviewers extensively searched in the electronic databases of Medline, Embase, Cochrane Central Register of Controlled Trials (CENTRAL), Web of Science, CINAHL, and Scopus for the studies published until the end of 2016. Only randomized clinical trials were included. The studied outcomes included FI episodes, FI score, resting pressure, squeezing pressure, and maximum tolerable pressure. The data were reported as Standardized Mean Differences (SMD) with 95% confidence interval.

**Results:**

Five articles were included in the present study (249 patients under treatment with PTNS and 239 in the sham group). Analyses showed that PTNS led to a significant decrease in the number of FI episodes (SMD=−0.38; 95% CI: −0.67–0.10; P=0.009). Yet, it did not have an effect on FI score (SMD=0.13; 95% CI: −0.49–0.75; P=0.68), resting pressure (SMD=0.12; 95% CI: −0.14–0.37; P=0.67), squeezing pressure (SMD=−0.27; 95% CI: −1.03–0.50; P=0.50), and maximum tolerable pressure (SMD=−0.10; 95% CI: −0.40–0.24; P=0.52).

**Conclusion:**

Based on the results, it seems that the prescription of PTNS alone cannot significantly improve FI.

## Highlights

- Posterior tibial nerve stimulation can lead to a significant decrease in the number of fecal incontinence episodes;
- Posterior tibial nerve stimulation have no effect on fecal incontinence score, resting pressure, squeezing pressure and maximum tolerable pressure;
- The prescription of posterior tibial nerve stimulation alone cannot significantly improve fecal incontinence.

## Plain Language Summary

Fecal Incontinence (FI) is a common and important disorder that affects more than 10% of adults. Although the operation is considered an essential treatment strategy in managing and treating FI, recently, a systematic review has indicated that there is not enough evidence to support operation as a clinical treatment in adults. Therefore, alternative or adjunctive treatments may be needed. Posterior Tibial Nerve Stimulation (PTNS) is an option for treatment that recent studies have indicated its high effectiveness. PTNS is low-cost and easy to use without anesthesia; however, there is still an ongoing controversy regarding PTNS in FI treatment. In this regard, the present systematic review and meta-analysis aimed at reaching a general conclusion regarding the role of PTNS in the control of FI. For this purpose, we searched for related papers indexed in Medline, Embase, Cochrane Central Register of Controlled Trials (CENTRAL), Web of Science, CINAHL, and Scopus databases. Finally, 5 articles were included in the present study. Analyses showed that PTNS led to a significant decrease in the number of FI episodes, but it had no effect on FI score, resting pressure, squeezing pressure, and maximum tolerable pressure. based on these results, it seems that the prescription of PTNS alone cannot lead to a significant improvement in FI.

## 1. Introduction

Pelvic floor disorders are currently regarded as one of the delicate problems worldwide since they not only cause health issues for the patient, but also in most cases they are not socially accepted and lead to the isolation of the patient. These problems harm the quality of life, and its high prevalence, especially in postpartum women, made it a social stigma (Biemans & van Balken, 2013).

Fecal Incontinence (FI) is a common and important disorder that affects more than 10% of adults (Rockwood et al., 2000; Rockwood et al., 1999). FI occurs because of i. anatomical defect such as obstetric and surgical rupture, congenital anomalies, and trauma or, ii. anal sphincter dysfunction, including rectum prolapse, constipation, neuropathy, and inflammatory bowel disease (Kamm, 1998). Primary treatments include conservative measures such as drug therapy and physiotherapy (pelvic floor training and biofeedback) (Madoff, Parker, Varma, & Lowry, 2004; Norton, Cody, & Hosker, 2006). Besides, surgical procedures (Madoff, 2004), the injection of biological agents (Malouf, Vaizey, Norton, & Kamm, 2001), artificial sphincter (Wong et al., 2002), and cell therapy (Sarveazad et al., 2017) like other tissues (Amini et al., 2016; Babahajian, Bahardoost, & Sarveazad, 2018; Babahajian, Shamseddin, & Sarveazad, 2017; Faghihi et al., 2015; Sarvandi et al., 2015; Sarveazad et al., 2017; Sarveazad et al., 2014; Yousefifard, Nasirinezhad, et al., 2016) are performed. Although the operation is considered an essential treatment strategy in managing and treating FI, recently, a systematic review has indicated that there is not enough evidence to support operation as a clinical treatment in adults. The study states that surgical methods are more invasive and accompanied by more serious side effects and without sufficient data on the long-term outcomes of this method (Forte et al., 2016). Therefore, alternative or adjunctive treatments may be needed.

Currently, the direct stimulation of pelvic nerves (such as sacral nerve) or indirect stimulation (such as of tibial nerve) is considered the second line of treatment in patients that have not responded to other approaches. Although the mechanism of nerve stimulation in FI management is unknown, the engagement of higher cortical centers and increased somatosensory representation of the anal region in the cortex, as well as higher consciousness of the brain regarding fecal continence, are possible mechanisms (George et al., 2012; Hotouras et al., 2012).

The stimulation of the sacral nerve was one of the first non-operation techniques used for the treatment of FI. Studies show that half of the patients will completely cure over 10 years (George et al., 2012; Matzel, Stadelmaie, Gall, & Hohenfellner, 1995). However, this method is not available in most clinics because of the high cost of the treatment and the need for high skill and experience of the therapist.

Posterior Tibial Nerve Stimulation (PTNS) is another option that recent studies have indicated its high effectiveness (Bondurri, Maffioli, & Danelli, 2015; de Groat & Tai, 2015; Edenfield, Amundsen, Wu, Levin, & Siddiqui, 2015; Giani & Musco 2015; Grossi et al., 2015; Gupta, Ehlert, Sirls, & Peters, 2015; Jiménez-Toscano et al., 2015; Kelly, Radley, & Brown, 2016; Lecompte, Hery, Guys, & Louis-Borrione, 2015; Lopez-Delgado et al., 2014; Marti & Maurus, 2014; Moya et al., 2016; Pena Ros et al., 2016; Sucar-Romero, Escobar-del Barco, Rodriguez-Colorado, & Gorbea-Chavez, 2014; Wexner, 2015). Although there is still an ongoing controversy regarding PTNS in FI treatment, PTNS is low cost and easy to operate without anesthesia (Hotouras et al., 2014; Hotouras et al., 2012). Therefore, a general conclusion is hard to reach at this time.

One way to overcome this problem is the performance of a systematic review and meta-analysis of the existing data. Although a systematic review was carried out in 2012 in this regard, it included only 7 studies. Researchers of the mentioned review claimed that the few numbers of studies, the presence of bias, and high heterogeneity in the existing data prevented reaching a conclusion about the effectiveness of PTNS in FI (Biemans & van Balken, 2013). Therefore, the present systematic review and meta-analysis aimed at reaching a general conclusion regarding the role of PTNS in the control of FI.

## 2. Methods

### Study design

The present study was designed based on the guideline of doing a meta-analysis of clinical trials in human studies (Cochrane). The data gathering tool in the present study was a checklist for summarizing the extracted data based on PRISMA guidelines. The method of doing the study and analyzing the data has been explained in detail in the previous meta-analyses of the researchers (Ahmadi & Yousefifard, 2017; Ebrahimi et al., 2014; Ghelichkhani et al., 2016; Hassanzadeh-Rad et al., 2016; Hosseini et al., 2017; Hosseini, Yousefifard, Aziznejad, & Nasirinezhad, 2015; Hosseini et al., 2015; Izadi, et al., 2016; Izadi et al., 2016; Mostafa, Mahmoud, Heidar, & Farinaz, 2015; Nakhjavan-Shahraki et al., 2017; Nakhjavan-Shahraki, Yousefifard, Oraii, Sarveazad, & Hosseini, 2017; Nakhjavan-Shahraki et al., 2018; Nasirinezhad, Hosseini, Karami, Yousefifard, & Janzadeh, 2016; Rahimi-Movaghar et al., 2016; Safari et al., 2016; Sarveazad et al., 2017; Yousefifard et al., 2016; Yousefifard, et al., 2016; Yousefifard et al., 2017).

### Search strategy

Two independent reviewers carried out an extensive search in the electronic databases of Medline, Embase, Cochrane Central Register of Controlled Trials (CENTRAL), Web of Science, CINAHL, and Scopus until the end of 2016. The search strategy was based on the words presented in Table 1. The keywords were selected to be as broad as possible to avoid missing any study. Although in the present meta-analysis, only human studies are included, the human filter was not applied to the search strategy to avoid mistakenly excluding a study. The applied keywords were selected, using MeSH from the PubMed database, EMTREE network of Embase database, and searching in the titles of the related articles found.

**Table 1.** Queries used in Medline, Embase, and Scopus databases

**Database**	**Search Terms**
Medline	(“fecal incontinence”[ MeSH] OR “fecal incontinence”[tiab] OR “fecal leak”[tiab] OR “faecal incontinence”[tiab] OR “Fecal Incontinences”[tiab]) AND (“tibial nerve”[MeSH Terms] OR “tibial nerve”[tiab] OR “Tibial Nerve”[tiab] OR “Tibial Nerves”[tiab] OR “Posterior Tibial Nerve”[tiab] OR “Posterior Tibial Nerves”[tiab] OR “tibial nerve stimulation”[tiab] OR “percutaneous tibial nerve stimulation”[tiab] OR “electrical nerve stimulation”[tiab] OR “nerve stimulation”[tiab] OR “Transcutaneous posterior tibial nerve stimulation”[tiab] OR “tibial neuromodulation”[tiab])
Embase	1- exp “fecal incontinence”/ or (“fecal incontinence” OR “Fecal leak” OR “faecal incontinence” OR “Fecal Incontinences”) ti, ab 2- exp “tibial nerve”/ or “tibial nerve” OR “Tibial Nerve” OR “Tibial Nerves” OR “Posterior Tibial Nerve” OR “Posterior Tibial Nerves” OR “tibial nerve stimulation” OR “percutaneous tibial nerve stimulation” OR “electrical nerve stimulation” OR “Nerve stimulation” OR “Transcutaneous posterior tibial nerve stimulation” OR “tibial neuromodulation”). ti, ab. 3- 1 & 2
Scopus	((TITLE-ABS-KEY (fecal incontinence) OR TITLE-ABS-KEY (fecal leak) OR TITLE-ABS-KEY (leak, fecal) OR TITLE-ABS-KEY (fecal incontinences) OR TITLE-ABS-KEY (incontinence, fecal))) AND ((TITLE-ABS-KEY (tibial nerve) OR TITLE-ABS-KEY (nerve, tibial) OR TITLE-ABS-KEY (tibial nerves) OR TITLE-ABS-KEY (posterior tibial nerve) OR TITLE-ABS-KEY (posterior tibial nerves) OR TITLE-ABS-KEY (tibial nerve stimulation) OR TITLE-ABS-KEY (percutaneous tibial nerve stimulation) OR TITLE-ABS-KEY (electrical nerve stimulation) OR TITLE-ABS-KEY (nerve stimulation) OR TITLE-ABS-KEY (transcutaneous posterior tibial nerve stimulation) OR TITLE-ABS-KEY (tibial neuromodulation)))

To find additional articles or unpublished data, we also did a hand-search in the list of relevant studies and related journals. In addition to search in the gray literature, 3 strategies were considered. First, a search was done in the section of the dissertation of the ProQuest database. Second, to find the unpublished or under-evaluation data, the authors of the related articles were contacted. Finally, Google and Google Scholar search engines were used to find additional references. In cases that the data could not be extracted, the article authors were contacted. In cases that the corresponding author did not respond to the first email, a reminder email was sent. If there were no response, another reminder email would be sent (with a one-week interval). If there were no response in the third stage, other authors of the article would be contacted, using social networks such as ResearchGate and LinkedIn to provide the researchers with the required data.

### Inclusion and exclusion criteria

In the present study, the randomized clinical trials that had evaluated the role of PTNS in FI were included. Controlled studies are defined as studies that have a control group without PTNS (operation alone or control group) in addition to the group that underwent PTNS. Adults (age>18 years old) were selected as the study population. The studies that had evaluated the motor function, ability to control bowel movements and anal sphincter pressure were included. Articles in which the researcher was not blind to the treatment group were excluded from the study. Besides, case report studies were excluded. The absence of a control or placebo group was also considered as another exclusion criterion.

### Quality assessment and data extraction

The results of the search in the literature were combined, and duplicate studies were removed, using End-Note (version X7, Thomson Reuters, 2015) software. In the second phase, during the primary screening, unrelated studies were eliminated. The abstracts of the extracted references were evaluated by 2 independent reviewers and recorded in the data extraction form and, if rejected, the reason would be mentioned. In case of disagreement between the 2 reviewers, the third reviewer studied the data and solved the disagreement by discussing. The extracted data included information regarding study design, sample characteristics, and control group (age, sex, and mechanism of FI), number of samples evaluated, outcome (FI scores and anal sphincter pressures), and probable biases. When the outcomes and studied measures were reported at various times, the last evaluation time was included in the study.

### Data synthesis

The evaluated outcomes consisted of FI episodes, FI scores, resting pressure, squeezing pressure, and maximum tolerable pressure. The data were summarized as mean and standard deviation and entered into the statistical software. The quality of studies was assessed using the guidelines suggested by Cochrane (Agency for Healthcare Research and Quality, 2012). To evaluate the agreement between the 2 reviewers, inter-rater reliability was appraised regarding the quality assessment of the studies (98%). In case of disagreement, it was solved by discussing it with the third reviewer.

### Data analysis

Statistical analysis was done with STATA version 14.0 (Stata Corporation, College Station, TX). Heterogeneity among the studies was evaluated using the Chi-square and I2 tests, and a P value of less than 0.1 was considered significant (showing heterogeneity). If the data were homogeneous, the fixed-effect model would be used. In cases that the cause of heterogeneity was not known, the random effect model was applied. Finally, the results of the studies were pooled, and the overall effect size was given. It is worth noting that meta-analysis was done when the associated data were reported by at least 3 studies. The funnel plot and Egger’s tests were applied for identifying publication bias (Egger, Smith, Schneider, & Minder, 1997).

## 3. Results

### Characteristics of the included studies

**Figure 1.**
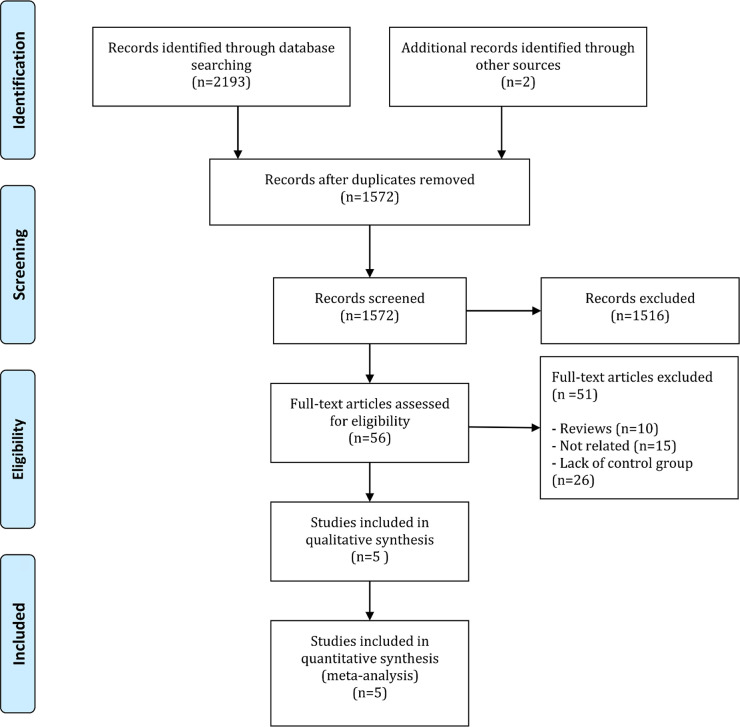
Flowchart of the present meta-analysis

During the initial search, after removing the duplicate articles, 1572 articles were screened. The full text of 56 articles was read. Finally, 5 articles were entered into the present study (Bouguen et al., 2014; George et al., 2013; Knowles et al., 2015; Leroi et al., 2012; Youssef et al., 2015) (Figure 1). These 5 articles included 6 independent experiments. One study (Leroi et al., 2012) had 2 separate experiments. All studies were clinical trials. Stimulation type was transcutaneous in 2 studies (Bouguen et al., 2014; Leroi et al., 2012), percutaneous in 2 (Knowles et al., 2015; Youssef et al., 2015), and both in 1 (George et al., 2013). These studies included the data of 488 patients; 249 patients were treated with PTNS, and 239 were in the control group. The mean age of the included patients was 56.6 years, and 73.0% of the patients were female. The studies had evaluated various outcomes. Resting pressure was evaluated in 4 experiments, squeezing pressure in 5 experiments, maximum tolerable pressure in 3 experiments, incontinence score in 5 experiments, and FI episodes in 4 experiments. Table 2 presents a summary of the data found in the included articles.

**Table 2.** Summary of included studies

Author, Year	Blinding	Stimulation Type	Stimulation Frequency	Session Duration (min)	Session Number	Sample size (control/ case)	Mean age (y)	Sex (male, %)	Assessed Outcome
Bouguen et al., 2014	Double blind	Percutaneous	10	20	300	9/10	64.7	5.26	Squeezing pressure
George et al., 2013	Single blind	Percutaneous and transcutaneous	20	30	50	8 / 22	57	6.67	FI episodes, incontinence scores, resting pressure, squeezing pressure, maximum tolerable volume
Knowles et al., 2015	Double blind	Transcutaneous	10	30	12	112/115	58	9.69	FI episodes, incontinence scores
Leroi et al., 2012	Double blind	Percutaneous	10	20	90	65/66	60	9.92	FI episodes, incontinence scores, resting pressure, squeezing pressure, maximum tolerable volume
Youssef et al., 2015	Single blind	Transcutaneous	10	20	12	37/36	44.2	54.79	Incontinence scores, resting pressure, squeezing pressure

FI=Fecal incontinence

### Risk of bias

**Figure 2.**
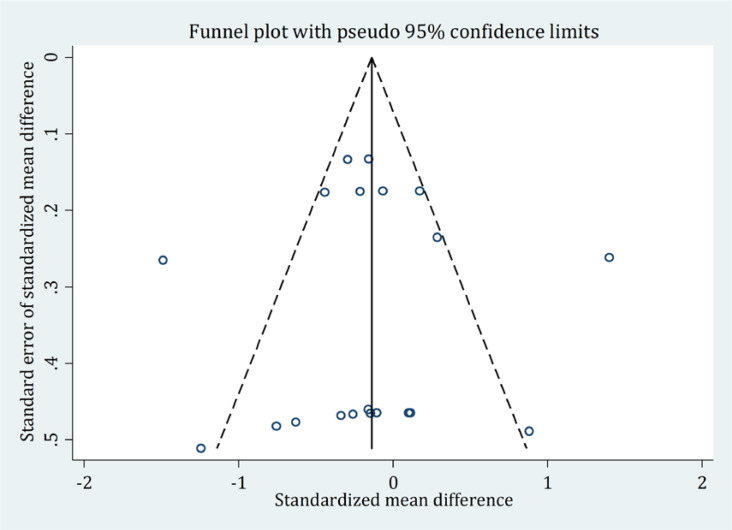
Assessment of publication bias based on the Egger test and Egger funnel plot

**Figure 3.**
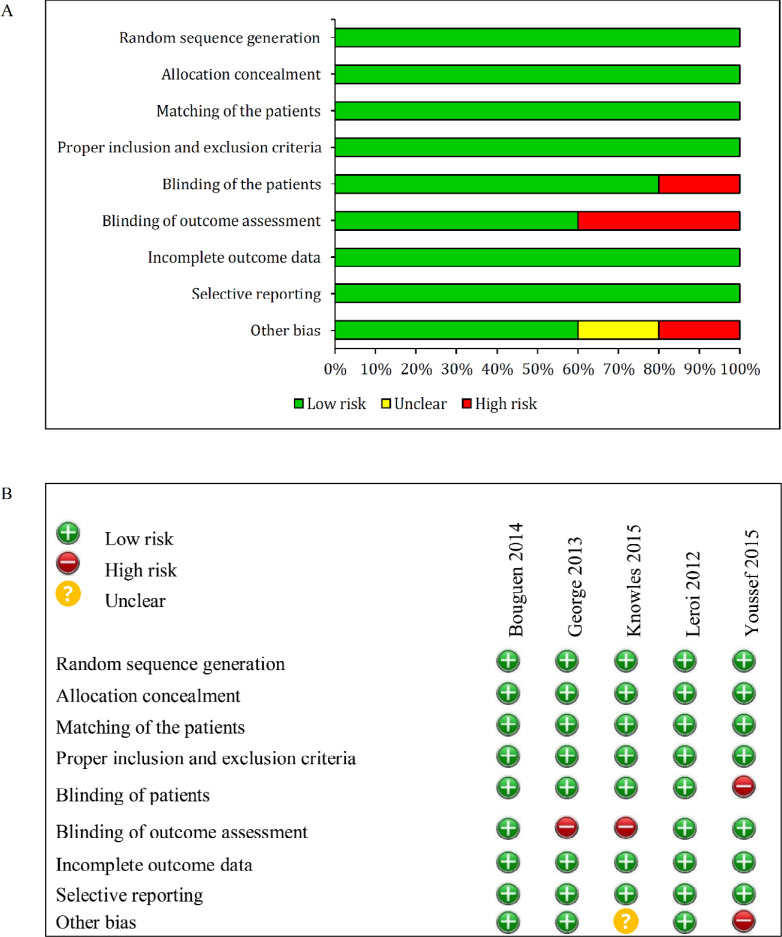
Risk of bias graph (A) and summary (B) in the included studies based on reviewers’ judgments

In the present study, there was no publication bias. According to Egger test (Coefficient=−0.06; P=0.96) and Begg’s test (Z=−0.8; P=0.40), no publication bias was detected (Figure 2). The risk of bias was low in all studies (Figure 3).

### Meta-analysis

#### FI episodes

**Figure 4.**
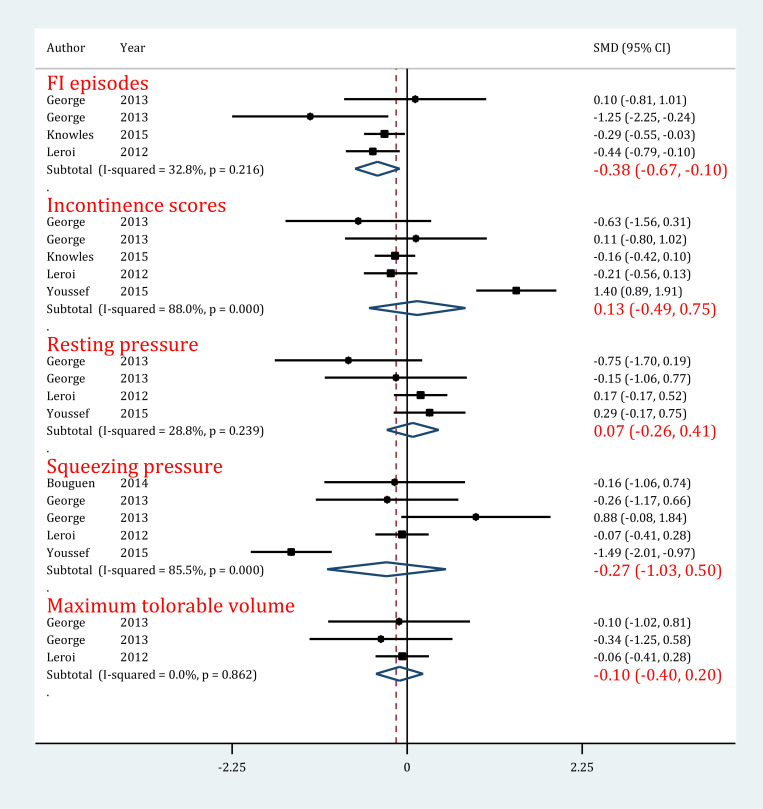
Forrest plot of administrating tibial nerve stimulation in the treatment of fecal incontinence Abbreviations: CI = Confidence Interval; SMD = standardized Mean Difference

In evaluating the effect of PTNS on FI episodes, 4 experiments were included (George et al., 2013; Knowles et al., 2015; Leroi et al., 2012). Heterogeneity was not present among the studies (I2=32.8%; P=0.22). Analyses showed that PTNS significantly decreased the number of FI episodes in patients with FI (SMD=−0.38; 95% CI: −0.67−0.10; P=0.009) (Figure 4).

#### Incontinence score

Five experiments were entered in this section (George et al., 2013; Knowles et al., 2015; Leroi et al., 2012; Youssef et al., 2015). The initial evaluation indicated the presence of significant heterogeneity among studies (I2=88.0%; P<0.0001). It should be noted that PTNS did not have any effect on the improvement of incontinence score in patients with FI (SMD=0.13; 95% CI: −0.49−0.75; P=0.68) (Figure 4).

#### Resting pressure

The data of 4 experiments were included in this section (George et al., 2013; Leroi et al., 2012; Youssef et al., 2015). It is worth mentioning that there was no heterogeneity in this regard (I2=28.8%; P=0.24). The findings showed that using PTNS does not have any effect on the improvement of resting pressure in patients with FI (SMD=0.12; 95% CI: −0.14−0.37; P=0.67) (Figure 4).

#### Squeezing pressure

Considering the data provided in 5 experiments (Bouguen et al., 2014; George et al., 2013; Leroi et al., 2012; Youssef et al., 2015), there was significant heterogeneity among the studies (I2=85.5%; P<0.0001). Analyses showed that using PTNS did not lead to more improvement in squeezing pressure of patients with FI compared to standard treatment (SMD=−0.27; 95% CI: −1.03−0.50; P=0.50) (Figure 4).

#### Maximum tolerable pressure

Three experiments were included in this section (George et al., 2013; Leroi et al., 2012) did not have heterogeneity (I2=0.0%; P=0.86). Like the previous 2 outcomes, PTNS did not have an effect on the improvement of maximum tolerable pressure in patients with FI (SMD=−0.10; 95% CI: −0.40−0.24; P=0.52) (Figure 4).

## 4. Discussion

In attempts to present less invasive treatment methods for FI, the subjects of neuromodulation, such as the stimulation of the pudendal nerve via an implant (Bock et al., 2010) and direct stimulation of anal sphincter (Herold, Bruch, Höcht, & Müller, 1988) were proposed, which showed little success. The stimulation of sacral plexus via stimulators is highly successful in treating FI; yet, its high costs (Klingler, Pycha, Schmidbauer, & Marberger, 2000) and the invasiveness of implanting the stimulator, which is associated with severe side effects (Brazzelli, Murray, & Fraser, 2006), are among the disadvantages of this method. Therefore, peripheral neuromodulation of sacral plexus, which is a non-invasive method, has been considered. Peripheral neuromodulation has been done since 1983 for the treatment of urinary incontinence, chronic pelvic pains, sexual disorders (McGuire, Zhang, Horwinski, & Lytton, 1983; Nakamura, Sakurai, Tsujimoto, & Tada, 1983; Vandoninck et al., 2003), and urologic cases, its success rate is the treatment with drugs and sacral nerve stimulation (Peters et al., 2009). It is explained by the indirect stimulation of the sacral plexus via the posterior tibial nerve, including sensory, motor, and autonomous fibers derived from the fourth and fifth lumbar and the first to the third sacral roots (Cooperberg & Stoller, 2005).

This systematic review aimed to find an answer to the question: Is PTNS useful for controlling FI or not? Perhaps the simplest mechanism that can be proposed for the effect of PTNS on FI is its effect on motor fibers that feed the anal sphincter, which results in the increased voluntary contractile power of the sphincter. This, in turn, can lead to an improvement in evaluating the measures of FI, including FI episodes and scores, maximum tolerable, and squeezing pressure. However, the result of our meta-analysis showed that PTNS can only be effective regarding FI episodes and has no effect on other measures. This finding indicates that various mechanisms are involved. These mechanisms are much more complicated than the direct effect in increased contractile power of sphincter.

Mechanisms by which PTNS improves FI are not fully understood, but in general, it can be said that PTNS leads to rectum sensory nerve stimulation (Michelsen, Buntzen, Krogh, & Laurberg, 2006; Rosen, Urbarz, Holzer, Novi, & Schiessel, 2001) and striated sphincter neuromodulation (Shafik, Ahmed, El-Sibai, & Mostafa, 2003). The effects of this neuromodulation decrease laxity of the anal canal and contraction in the rectum (Shafik et al., 2003; Vaizey, Kamm, Turner, Nicholls, & Woloszko, 1999), increase bloodstream in the rectal mucosa (via stimulation of autonomous fibers) (Emmanuel & Kamm, 1999), and change the central neurotransmitter environment (Chang et al., 1998; Cooperberg & Stoller, 2005). The PTNS mechanisms of action in FI can be evaluated in 2 levels: i. central nervous system and, ii. peripheral nervous system. In the central mechanism, since the alternating stimulation of posterior tibial nerve leads to the inhibition of Aδ pain afferent fibers in the spinothalamic tract (Chung, Lee, Hori, Endo, & Willis, 1984), it is thought that pathologic sensory information (in abnormal passing of urine and stool) and the consciousness of the pathological situation are inhibited. Therefore, central nervous system responses regarding bowel disorders are adjusted (Bemelmans, Mundy, & Craggs, 1999; de Groat, 1997).

The peripheral mechanism of PTNS is mediated via nerves that originate from the sacral plexus, which innervate the anorectal region. In a mixed nerve (such as the pudendal nerve), the stimulation threshold of various fibers is different. The lowest threshold belongs to the motor fibers of Aα and the highest to C fibers (transmission of pain). Therefore, when stimulating via an implant, the weakest stimulation activates alpha motor fibers. Then, reflexes are produced after the activation of Aδ fibers, which have a higher threshold. In weak stimulations (like superficial stimulation produced by electrodes during PTNS), although initially Aα motor fibers that feed the external anal sphincter are stimulated, this stimulation is not enough for increasing the pressure of the external anal sphincter and this can be a logical explanation for the result of our study, indicating that PTNS does not affect squeezing pressure (squeezing pressure is 100% related to the contractile power of the external anal sphincter (Bansal, Sachdeva, Jain, Ranjan, & Arora, 2013). However, this stimulation may be enough for activating Aδ reflexes (Vaizey et al., 1999). Therefore, enhancing and adjusting these reflexes following PTNS can be a logical explanation for the findings in our study, indicating a decrease in FI episodes. These reflexes include Anorectal Tightening Reflex (ATR) and Gower’s reflex.

During the slow filling of the rectum ATR by the contraction of the internal anal sphincter and during rapid rectal filling Gower’s reflex via the contraction of the external anal sphincter leads to fecal continence (Shafik, 1993). As mentioned before, sensory fibers such as pain have the highest stimulation threshold for activation; therefore, they are not activated in weak stimulations (Vaizey et al., 1999). As a result, it is no surprise that as the findings of our study show, PTNS does not affect the maximum tolerable volume, which indicates rectal sensation (Kim, 2010).

To sum up, the present study had some strong points and weak points. Among the strong points of the study were extensive search in literature, doing a hand search repeatedly contacting the authors for obtaining information related to the included articles. Another strong point of the current study was including only randomized clinical trials that had a control group. This resulted in; first, a good level of quality of the included trials and, second, minimizing publication bias and heterogeneity among the studies. However, the present study also had some limitations. One of the most important limitations was the inability to do subgroup analysis because of the small number of the included articles. The second limitation of this study was the presence of 2 single-blind studies among 5 studies that were evaluated, which might have caused the bias and be the source of the heterogeneity seen in some of the studied outcomes. Since the subgroup analysis was absent, the effect of variations in blinding the studies on the results could not be assessed.

## 5. Conclusion

The present meta-analysis attempted to reach a conclusion from the existing studies regarding the effectiveness of PTNS in the treatment of FI. The findings of the present study showed that PTNS only decreased FI episodes, but did affect anal sphincter pressure and FI scores. Therefore, based on these results, it seems that the prescription of PTNS alone cannot lead to a significant improvement in FI.

## Ethical Considerations

### Compliance with ethical guidelines

All ethical principles were considered in this article.
